# Minimum Effects of Sampling Time on the Apparent Digestibility of Nutrients and Blood Protein Catabolites in Light Lambs

**DOI:** 10.3390/ani11082244

**Published:** 2021-07-30

**Authors:** Jonathan Pelegrin-Valls, Beatriz Serrano-Pérez, Daniel Villalba, Ester Molina, Javier Álvarez-Rodríguez

**Affiliations:** 1 Departamento de Ciencia Animal, Universidad de Lleida, Av. Alcalde Rovira Roure 191, 25198 Lleida, Spain; beatriz.serrano@udl.cat (B.S.-P.); daniel.villalba@udl.cat (D.V.); ester.molina@udl.cat (E.M.); javier.alvarez@udl.cat (J.Á.-R.); 2 AGROTECNIO-CERCA Center, Av. Alcalde Rovira Roure 191, 25198 Lleida, Spain

**Keywords:** circadian, crude protein, blood catabolites, sheep

## Abstract

**Simple Summary:**

Nowadays, animal nutritionists are more aware of how expensive and limited protein is in animal feedings. Understanding the role of sampling time in evaluating the nutritional status of small ruminants could be helpful to assess more efficiently dietary crude protein content. However, faeces or blood samples to analyse dietary changes are taken at indistinct times of the day, depending on the availability of the technicians. Thereby, this study was designed to evaluate the effect of sampling time (8:00 a.m., 12:00 p.m., and 4:00 p.m.) on some apparent nutrient digestibility and blood catabolites related to nutritional status. Our findings revealed that one sample of faeces or blood in the morning enables the optimal evaluation of the impact of crude protein variations in diet formulations.

**Abstract:**

This experiment aimed to evaluate the effects of sampling time on organic matter (OM), crude protein (CP) and phosphorous (P) apparent digestibility and plasma urea and creatinine concentration in growing and finishing male Ripollesa lambs fed different CP concentrations in the diet. Twenty-four male Ripollesa lambs with 14.5 kg body weight (BW) were randomly assigned to two groups differing in CP content in the growing (14 to 19 kg of BW) and finishing (19 to 25 kg of BW) phases (20% vs. 18% CP and 19% vs. 17% CP, respectively). Faeces collected from the rectum and blood samples collected from the jugular vein were taken at 8:00 a.m., 12:00 p.m., and 4:00 p.m. During the growing period, the OM, CP and P apparent digestibility were higher in the lower CP diet (*p* < 0.05), but only P was affected by the sampling time, being highest at 8:00 a.m. (*p* < 0.05) compared to other sampling hours. During the finishing period, there were no differences in these digestibility coefficients between diets or sampling times (*p* > 0.05). Sampling time did not affect (*p* > 0.05) plasma urea concentrations either in the growing or finishing period. Plasma creatinine concentrations did not differ (*p* > 0.05) between lambs receiving 18% or 20% CP diets, but during the finishing period, it was lower at 4:00 p.m. in lambs fed 17% CP (*p* < 0.05) than those offered 19% CP. Overall, the results suggest that the collection schedule to evaluate the protein nutritional status can be shortened through one spot sample of faeces or blood in the morning.

## 1. Introduction

Spain is the top sheep producer within the European Union [1]. In this country, the most common production system is a light lamb weaned at 30–60 days of age with 12–15 kg of body-weight (BW) and raised subsequently on a concentrate diet ranging from 15% to 21% crude protein (CP) on a dry matter (DM) basis [2] until 75–100 days of age with 24–28 kg of BW. This intensive feeding system could result in a high environmental load of nutrients, such as nitrogen and phosphorous (P). Therefore, these nutrients need to be adjusted not only to reduce their excretion and subsequent potential pollution on the environment, but also because high protein diets are very demanding of protein-rich ingredients, which have been demonstrated to boost the carbon footprint of livestock production [3], and also because P is a finite and non-renewable resource [4]. Despite significant efforts for adjust these nutrients in diets for livestock, there is still the need to improve feeding animal practices to reduce manure nitrogen and P in intensive sheep production systems [5].

Animal nutritionists need to know how changes in the lambs’ diet affect their metabolism and feed digestibility in commercial flocks. Therefore, to optimise the assessment of dietary CP in lamb’s nutrition, it is necessary to develop a better knowledge of diurnal variation on nutrient apparent digestibility and blood metabolites related to nutrient status. Although urinary nitrogen could be helpful when dietary CP is studied, blood urea and creatinine have a more significant potential for utilisation because of their reliable reflection in the blood [6]. In this regard, blood urea concentration is used to evaluate the protein status of ruminants [7] as it is linked to protein catabolism [8], and it does not differ during daylight in growing calves fed ad libitum [9]. On the other hand, blood creatinine concentration reflects a loss of skeletal muscle mass [10], but it may be affected by time of the day.

Regarding apparent digestibility, Fukumoto et al. [11] concluded that only one daily faecal sample collection between 9 h and 13 h is needed to estimate the digestibility of nutrients in sheep fed ad libitum. The use of internal markers, such as the acid-insoluble ash (AIA) technique, to determine the digestibility of nutrients from faecal samples collected during the day has been uniformly accepted [12], assuming that AIA is a natural component of feeds that is expected to flow with the digesta through the gastrointestinal tract of the animal. Additionally, it has been accepted that markers can be used throughout the faecal collection period for recording digestibility. However, the natural event of transit and degradation of ingested feed, although continuous in the rumen, may not be constant throughout the remainder of the digestive tract of ruminants [13]. Even though Keulen and Young [14] showed no evidence of a diurnal variation in AIA excretion in sheep, a potential interaction between sampling time and diet composition may not be discarded, since Morris et al. [15] observed in ruminants a diurnal variability in AIA excretion when two forage diets were compared. This suggests that diet composition may influence the digestive process of marker excretion.

Hence, this study aimed to use a single faecal or blood sample to test the apparent digestibility of nutrients and blood metabolites related to protein status in growing and finishing lambs fed different CP concentration levels.

## 2. Materials and Methods

### 2.1. Animals, Diets and Experimental Design

The experiment was carried out in the experimental facilities of El Nial of the BonÀrea Agrupa (Guissona, Lleida, Catalonia, Spain, 41°46′32.2″ N, 1°16′33.2″ E; 484 m above sea level) between January and February 2018. A total of sixty weaned (45–60 days-old) male Ripollesa lambs weighing 14.5 ± 1.3 kg were housed in 12 shared pens (5 animals/pen; 1.04 m^2^ per animal) and they were distributed in homogeneous groups according to their initial BW. Twenty-four lambs were randomly selected from the whole flock for this study (2 lambs/pen). In each pen, lambs had access to one longitudinal multiplace feeder with barley straw and a creep concentrate feeder. Lambs were fed in two periods according to their BW, the growing (14 to 19 kg BW) and finishing (19 to 25 kg BW) period, which lasted 21 days each. Four experimental diets with different CP levels were formulated and supplied to two treatment groups: half of the lambs (*n* = 12) were fed a diet containing 20.8% CP (CP20 group) and 19.1% CP (CP19 group) on dry matter basis (DM) during the growing and finishing periods, respectively, whereas the other half (*n* = 12) were fed diets containing 18.3% CP (CP18 group) and 17.4% CP (CP17 group) on a DM basis during the growing and finishing periods, respectively. Before the experiment started, lambs were fed standard commercial diets for five days adaptation concentrate containing coccidiostatic (decoquinate at 30 mg/kg). Ingredients and chemical composition of the experimental diets can be found in Pelegrin-Valls et al. [16], and the organic matter (OM), CP, P and AIA contents are reproduced in Table 1. Briefly, pelleted diets were isoenergetic (1760 kcal of Net Energy for Ruminants/kg of concentrate) and they were formulated with the same ingredients and additives in the same manufacturing batch. Only the percentage of vegetable protein was modified. In both periods, lambs had free access to concentrate, water and barley straw.

### 2.2. Sampling

The offered concentrate and straw were recorded daily, and the refused straw and concentrate were recorded once weekly on a pen basis. Thereby, cumulative feed disappearance was recorded, and feed intake was considered steady for the whole week. The concentrate was offered every morning in the creep feeder. Lambs were individually weighed once a week to calculate average daily gain (g/day) by regression of BW on time. In the last weeks of both the growing and finishing periods, faeces pools of approximately 50 g, coming from at least 3 lambs per pen (6 pen replicates/dietary treatment), were collected at 8:00 a.m., 12:00 p.m., and 4:00 p.m. by rectal stimulation to determine apparent digestibility coefficients of OM, CP and P. Samples of concentrate and straw were also collected at 8:00 a.m. All feed samples were kept at −20 °C until analysis. After thawing, samples were weighed and dried in a forced air stove at 60 °C for 72 h. The moisture of samples was determined by the weight difference between the fresh and DM. After drying, samples were ground in a knife mill to pass a 1 mm sieve and stored in watertight plastic bags until analysis. The total digestive tract apparent OM, CP and P digestibilities were calculated using the nutrient-to-marker ratio in the diet and faeces, as follows:

Apparent digestibility coefficient (%) = 100 − [100 × (Marker_diet_/Marker_faeces_) × (Z_faeces_/Z_diet_)] (1)

where Z_faeces_ and Z_diet_ are the nutrient concentrations (%) in faeces and diet, respectively. The Marker_faeces_ and Marker_diet_ are the concentrations (%) of AIA in faeces and diet, respectively. The Z_diet_ and Marker_diet_ were calculated by considering the amount of concentrate and straw consumed per pen. The recovery rate of AIA in faeces was assumed to be complete [12].

At the same time of faeces collection, blood samples were collected from two lambs per pen at 8:00 a.m., 12:00 p.m., and 4:00 p.m. The same lambs, that were randomly selected at the start of the trial, were sampled during the day and at both rearing periods. Vacuum tubes with EDTA (BD Vacutainer^®^, Becton, Dickinson and Company, Plymouth, UK) were used to collect 5 mL of blood from the jugular vein (6 pen replicates /dietary treatment) and were centrifuged in situ at 3000× *g* for 10 min to obtain the plasma, which was stored in identified aliquots for each sampled lamb at −20 °C until metabolites analysis.

### 2.3. Chemical Analyses

The ash content to calculate OM was determined with 2 g of feed and faeces samples in a muffle furnace at 550 °C for 3 h. CP content of diets (N × 6.25) was determined following the DUMAS procedure, using a nitrogen and protein analyser (Model NA 2100, CE Instruments, Thermoquest SA, Barcelona, Spain). P was determined by ultraviolet-visible spectroscopy (ICP-OES, HORIBA Jobin Yvon, Activa family, with AS-500 Autosampler, HORIBA Scientific, Madrid, Spain). Apparent OM, CP and P digestibility coefficients were estimated by the AIA technique, which was determined following the procedure described by Álvarez-Rodríguez et al. [17]. Feed and faeces samples were analysed in duplicate.

Plasma urea and creatinine concentration (mg/dL) were analysed by an automatic analyser (GernonStar, RAL/TRANSASIA, Dabhel, India). The kinetic method was used to quantify plasma urea which catalysed the hydrolysis of urea into ammonia and carbon dioxide. The test had a measurement range between 2 and 350 mg/dL and their mean intra- and inter-assay coefficients of variation were 2.8% and 2.7%, respectively. Plasma creatinine was quantified using the enzymatic method as final by-product of the muscular metabolism. The creatinine measurement range was 0.03 to 50 mg/dL with mean intra- and inter-assay coefficients with variations of 3.1% and 5.1%, respectively.

### 2.4. Statistical Analysis

The cumulative concentrate and straw intake, in the growing and finishing periods, were analysed with standard least square means models with dietary treatment as fixed effect. Plasma metabolites and nutrient digestibility data, in each growing and finishing periods, were analysed with the statistical software JMP Pro13 (SAS Institute Inc., Cary, NC, USA), using mixed models with repeated measurements that included the dietary treatment, the sampling time and their interaction as fixed effects, and the pen as a random effect. Results are presented as least square means and their standard error. The comparison of means was carried out with the Tukey′s test. The level of significance was set at 5%.

## 3. Results

### 3.1. Animal Performance, Dietary and Faecal AIA Content and DM of Faeces

The average concentrate and straw intake in the growing period did not differ (*p* > 0.05) between CP20 and CP18 groups (686 vs. 753 ± 27.3 g and 104 vs. 97 ± 7.3 g, respectively). In the finishing period, CP19 and CP17 groups also had a similar concentrate and straw intake (812 and 843 ± 16.7 g and 120 vs. 117 ± 8.5 g, respectively; *p* > 0.05). The overall forage to concentrate ratio was 13:87 and 12:88 for the growing and finishing periods, respectively. The average daily gain did not differ between CP20 and CP18 diets either in the growing period (216 vs. 237 ± 17.9 g/day, respectively; *p* > 0.05), finishing period (296 vs. 272 ± 23.7 g/day, respectively; *p* > 0.05) or overall period (242 vs. 259 ± 12.0 g/day, respectively; *p* > 0.05).

Concentrate and straw AIA contents were analysed only from samples at 8:00 a.m. as it was considered that their contents did not change between hours of sampling. For the growing and finishing periods, the dietary AIA content was greater for the CP20 group than CP18 group and for the CP19 group than CP17 group (0.86 vs. 0.79 ± 0.01% and 0.87 vs. 0.77 ± 0.01%, respectively; *p* < 0.05).

Results of faecal composition are shown in Table 2. DM of faeces was similar for CP20 and CP18 group during the growing period, and in the CP19 and the CP17 group during the finishing period (Table 2; *p* > 0.05).

In the growing period, no significant differences were observed between the sampling times for DM of faeces. However, in the finishing period, DM of faeces was lower at 8:00 a.m. than at 12:00 p.m., and 4:00 p.m. (Table 2; *p* < 0.05).

OM and CP in the faeces content did not differ between treatments and sampling times (Table 2; *p* > 0.05). Nevertheless, faecal P content during the growing period was higher in the CP20 than in the CP18 group (Table 2; *p* < 0.05), although these differences were not distinguished in the finishing period. Similarly, in the sampling times, less P was observed in the faeces at 8:00 a.m. compared to 12:00 p.m. and 4:00 p.m. (Table 2; *p* < 0.05), but not in the finishing period.

The faecal AIA content in the growing period was lower in CP20 than CP18 groups (*p* < 0.05). However, in the finishing period, no differences were observed between the dietary treatments in faecal AIA content (Table 2; *p* > 0.05).

The faecal AIA content did not differ across sampling times in any period (Table 2; *p* > 0.05).

### 3.2. Nutrient Digestibility and Blood Metabolites

**Figure 1:**
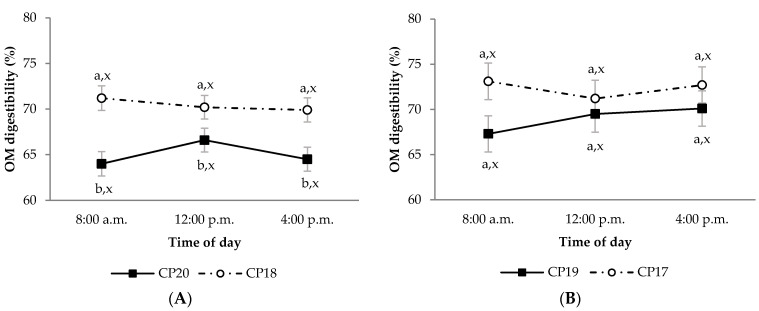
Digestibility of organic matter (OM) in growing (**A**) and finishing (**B**) periods in Ripollesa lambs fed with different concentrations of crude protein were compared between treatments (CP20 vs. CP18 and CP19 vs. CP17) and time of the day (least square mean values ± standard error). Within each sampling hour, different letters (a, b) denote statistical differences (*p* ≤ 0.05) between dietary treatments. Within each dietary treatment, different letters (x, y) denote statistical differences (*p* ≤ 0.05) between sampling hours.

**Figure 2:**
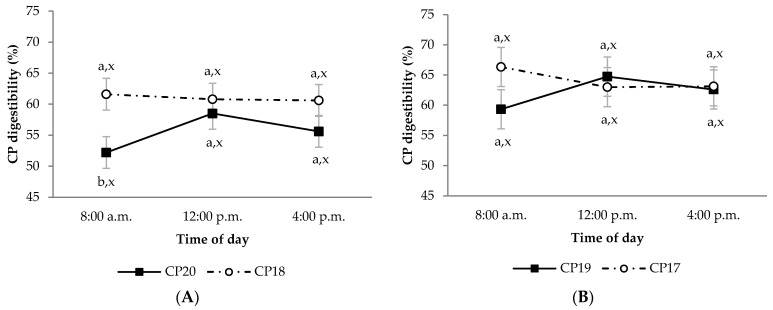
Digestibility of crude protein (CP) in growing (**A**) and finishing (**B**) periods in Ripollesa lambs fed with different concentrations of crude protein were compared between treatments (CP20 vs. CP18 and CP19 vs. CP17) and time of the day (least square mean values ± standard error). Within each sampling hour, different letters (a, b) denote statistical differences (*p* ≤ 0.05) between dietary treatments. Within each dietary treatment, different letters (x, y) denote statistical differences (*p* ≤ 0.05) between sampling hours.

**Figure 3:**
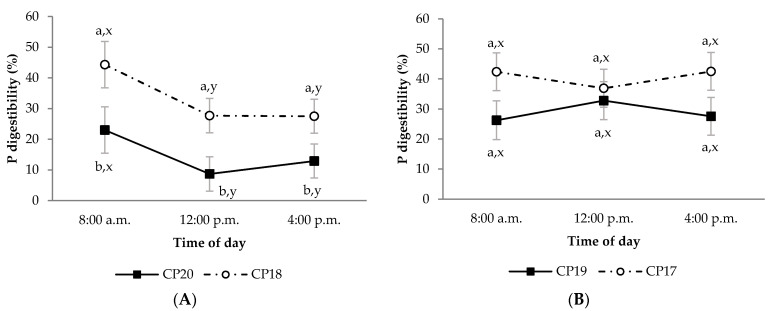
Digestibility of phosphorous (P) in growing (**A**) and finishing (**B**) periods in Ripollesa lambs fed with different concentrations of crude protein were compared between treatments (CP20 vs. CP18 and CP19 vs. CP17) and time of the day (least square mean values ± standard error). Within each sampling hour, different letters (a, b) denote statistical differences (*p* ≤ 0.05) between dietary treatments. Within each dietary treatment, different letters (x, y) denote statistical differences (*p* ≤ 0.05) between sampling hours.

**Figure 4:**
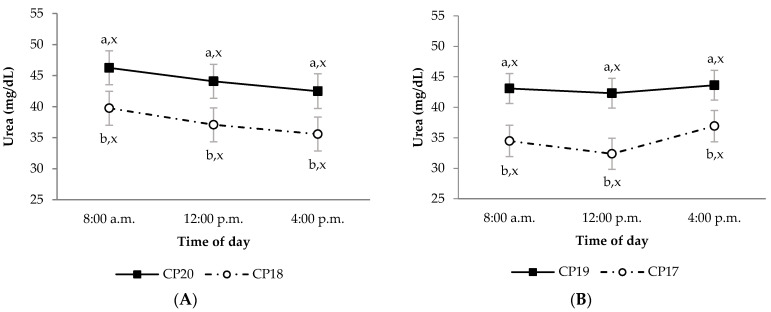
Plasma urea concentrations (mg/dL) in growing (**A**) and finishing (**B**) periods in Ripollesa lambs fed with different concentrations of crude protein were compared between treatments (CP20 vs. CP18 and CP19 vs. CP17) and time of day (least square mean values ± standard error). Within each sampling hour, different letters (a, b) denote statistical differences (*p* ≤ 0.05) between dietary treatments. Within each dietary treatment, different letters (x, y) denote statistical differences (*p* ≤ 0.05) between sampling hours.

**Figure 5:**
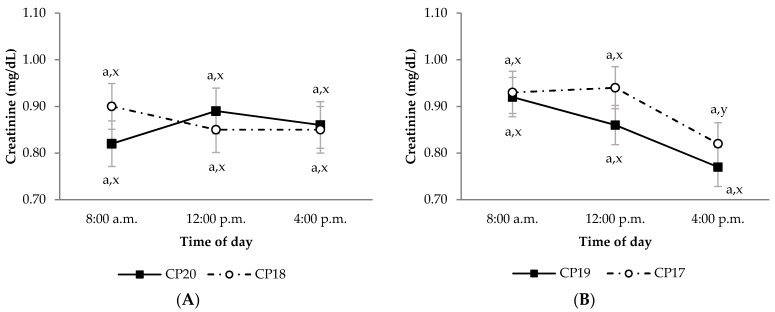
Plasma creatinine concentrations (mg/dL) in growing (**A**) and finishing (**B**) periods in Ripollesa lambs fed with different concentrations of crude protein were compared between treatments (CP20 vs. CP18 and CP19 vs. CP17) and time of day (least square mean values ± standard error). Within each sampling hour, different letters (a, b) denote statistical differences (*p* ≤ 0.05) between dietary treatments. Within each dietary treatment, different letters (x, y) denote statistical differences (*p* ≤ 0.05) between sampling hours.

During the growing period, diet OM digestibility in the CP20 group was lower than in CP18 group, regardless of sampling time (Figure 1A; *p* < 0.05). This effect was also observed in the CP20 group compared to CP18 group for diet CP apparent digestibility, but only at 8:00 a.m. (Figure 2A; *p* < 0.05). Moreover, the CP20 group had lower diet P apparent digestibility compared to CP18 group (Figure 3A; *p* < 0.05). On the contrary, plasma urea concentrations were greater in CP20 than CP18 group (Figure 4A; *p* < 0.05), but no differences were observed in plasma creatinine concentrations (Figure 5A; *p* > 0.05). The blood urea/creatinine ratio was also different between dietary treatments studied (51.9 vs. 43.9 ± 2.3 for CP20 and CP 18, respectively, *p* < 0.05).

Sampling time did not affect (*p* > 0.05) apparent digestibility of OM and CP in any treatment during the growing period (Figure 1A and Figure 2A). However, sampling time affected the apparent digestibility of P, which was greater at 8:00 a.m. (*p* < 0.05) compared to 12:00 p.m. and 4:00 p.m. (Figure 3A). On the other hand, urea and creatinine concentrations at different sampling times were similar (*p* > 0.05) between dietary treatments (Figure 4A and Figure 5A). Likewise, the plasma urea/creatinine ratio was similar between sampling hours (50.7 at 8:00 a.m., 46.9 at 12:00 p.m. and 46.1 ± 2.07 mg/dL at 4:00 p.m.; *p* > 0.05).

During the finishing period, the OM, CP and P apparent digestibility was similar between CP19 and CP17 groups (Figure 1B, Figure 2B and Figure 3B, respectively; *p* > 0.05). The CP19 group had a higher plasma urea concentration than CP17 group (Figure 4B; *p* < 0.05), nevertheless, plasma creatinine concentrations were similar between dietary treatments (Figure 5B; *p* > 0.05). On the contrary, plasma urea/creatinine ratio was higher in CP19 than in CP17 (51.4 vs. 39.6 ± 2.3, respectively, *p* < 0.05).

No differences existed between sampling times for OM, CP and P apparent digestibility in any of the groups studied during the finishing period (Figure 1B, Figure 2B and Figure 3B, respectively; *p* > 0.05). Likewise, sampling times did not affect plasma urea concentrations for any dietary treatment (Figure 4B; *p* > 0.05), but the CP17 group had higher plasma creatinine concentrations at 8:00 a.m. and 12:00 p.m. than at 4 p.m. (Figure 5B; *p* < 0.05), while no differences between sampling times were observed in the CP19 group (*p* > 0.05). In turn, the urea/creatinine ratio was lower at 8:00 a.m. and 12:00 p.m. than at 16:00 p.m. (42.6 and 42.7 vs. 51.5 ± 2.42 mg/dL, respectively; *p* < 0.05).

## 4. Discussion

The objective of this study was to determine the effect of sampling time on the apparent nutrient digestibility and blood metabolites related to nutritional status in Ripollesa light lambs fed different levels of CP. Most of the evaluated variables did not show interaction between daily sampling time and dietary CP, and thus these effects are discussed separately.

### 4.1. Dietary CP Effect on Nutrient Digestibility and Plasma Metabolites

In a previous study, we reported that CP content of lamb feed can be reduced (10% below the current commercial standards) without a negative effect on growth performance or carcass yield of local Spanish breeds [16]. However, the impact of a reduction in the amount of dietary protein on metabolism should be carefully evaluated and adequate methods are needed, as many metabolic processes in mammals have been described as being influenced by eating frequency [18], which could impair the proper metabolic synthesis of proteins, and consequently, the growth of lambs.

In this study, a reduction in dietary CP during growing and finishing periods did not affect the faecal consistency, as DM of faeces remained steady. However, the dietary AIA content was greater in lambs fed high CP diets in the growing period. Probably, these group ingested a little more straw, but these mild differences were not supported by statistical differences in total feed intake. To estimate the apparent digestibility of nutrients, the AIA marker is more accurate when the dietary AIA is higher than 0.75% (DM basis) [19]. This was indeed attained by both dietary treatments in the growing and finishing periods with an intensive concentrate inclusion (nearly 90% of the ration) and barley straw supplement. The reliability of internal markers such as AIA has been questioned as it occasionally overestimates digestibility coefficients when compared with other markers [15,17]. However, Pepeta et al. [20] found that AIA could be used as an accurate and precise marker for estimating nutrient digestibility in sheep. To use AIA as digestibility marker in concentrate-based feeding systems, it is essential to supplement lambs’ with roughages, as barley straw, at dietary levels >10%. Therefore, to accurately estimate the nutrient digestibility through internal markers, it is necessary to take into account the feeding frequencies and dietary components [21].

The role of reducing the CP in the lambs’ diet on the digestive efficiency may be evaluated through the apparent digestibility of nutrients. Accordingly, during the growing period, it was found that animals fed the CP18 diet had better OM digestibility. Similar results were obtained by Haddad et al. [22], who observed that heavy lambs improved OM digestibility when fed diets containing CP level was less than 18%. In addition, bone (rich in P) and lean (rich in CP) tissues had early developing allometric growth coefficients (b < 1) in relation to carcass weight of lambs [23]. Thereby, these two tissues are mostly developed during the growing period of light lambs, which may support the differences in CP and P apparent digestibility between dietary CP levels in this stage. However, this improvement in the faecal apparent OM digestibility with the reduction in dietary CP was not observed during the finishing period.

The P apparent digestibility in the growing period was lower in the CP20 group than in CP18 group. Borges et al. [24] noted that P digestibility depends on its diet content, nutrient sources and physiological status. Furthermore, it has been described that faeces are the principal pathway of P excretion in ruminants, and it is directly correlated with the diet P content [24]. However, according to Dias et al. [25], the faecal loss of P may be overestimated because of salivary P secretion, suggesting that the true available P was probably greater in both dietary treatments. Assuming that the urinary loss of this mineral is minimal, the metabolic P requirements may be related to the mineral proportion of skeletal body weight gain [26]. As these lambs had similar average daily gain, the greater P apparent digestibility in CP18 lambs compared to CP20 lambs may be the result of a slightly higher dietary concentration of this nutrient (0.45% vs. 0.42%, respectively) and a lower salivary P secretion to buffer rumen pH conditions, which can represent as much as 80% of the endogenous secretion in the rumen [24]. On the contrary, in the finishing period, no differences were observed in OM, CP and P apparent digestibility between experimental diets, which suggests that the CP17 group met its nutritional requirements to a greater extent.

Plasma urea concentration was greater in lambs fed the highest CP diets. This is in line with Mahmoud et al. [27] who concluded that heavy lambs fed diets with high CP levels had greater plasma urea concentrations than those fed lower CP levels. Plasma urea concentration reflects the amount of protein ingested and thus the absorption of ruminal ammonia [28]. Hence, the reduction of dietary CP decreases the protein catabolism. However, plasma creatinine concentrations showed no differences between dietary treatments and sampling periods. In this regard, the current results suggest that lower CP diet did not promote muscle degradation because of insufficient dietary protein [29].

### 4.2. Sampling Time Effect on Nutrient Digestibility and Plasma Metabolites

It has been reported that feed intake of lambs is reduced at night as a result of an instinctive fear of predation [30], so it would result in less excretion of faeces early in the morning. On the other hand, according to Sampaio et al. [13], feed degradation and transit in the rumen are continuous but influenced by actual feed intake. These authors observed that feed degradation and faecal elimination increase during feed intake periods, which causes more inconsistency in the faecal content. Additionally, two peaks of eating and drinking occur in feedlot lambs [31] and grazing lambs [17], which occur near sunrise (about 8:00 a.m.) and sunset (about 8:00 p.m.). The faecal sampling schedule was planned to gather the potential digestive turnover differences after the morning eating time under ad libitum feeding conditions. During the finishing period, faeces DM was lower at 8:00 a.m. However, this variation did not have any relationship with the outcomes of digestibility coefficient calculations throughout the daylight, as discussed below.

A lack of representativity in the collection of the faecal samples may cause an estimation bias in the digestibility estimates [13]. In this regard, only a slight variation in diurnal faecal excretion of the AIA marker has been seen over several days in rabbits [32]. Similarly, Kanani et al. [33] reported that cattle were not affected by sampling time and there was no diet by sampling time interactions, even though diet affected faecal AIA concentrations, which is in agreement with the present outcomes in sheep.

The sampling time in the light lambs of the present study showed no effect on OM and CP apparent digestibility in any of the periods studied. This would mean that the marker digestibility estimations were uniformly distributed throughout the entire faecal sample collection period. Similar results were obtained by Paternostre et al. [34] in growing pigs, who concluded that only one spot-sampling was enough (either at 9:00 a.m. or 2:00 p.m.) to estimate the faecal OM digestibility by markers in animals fed ad libitum. On the contrary, in the growing period, P apparent digestibility was greater early in the morning (8:00 a.m.) while in the finishing period, this difference did not persist. As stated earlier, faecal P may include much of the P secreted by saliva that is not reabsorbed [35], and early morning faecal samples may reflect an increase in undigested P that would be linked to increased salivary P turnover, which is induced by rumination that is usually carried out at night [30].

Plasma urea concentrations showed no differences between sampling times in any of the periods studied. Piccione et al. [36] found, in sheep, that both salivary and blood urea profiles were high during the light phase and low during the dark phase of the natural light–dark cycle. Within 12 h after feeding, Valkeners et al. [37] observed minimal variations in blood urea concentration in calves, which in turn showed a 3 h delay after ruminal ammonia synthesis. In this regard, Oliveira et al. [38] described lower rumen ammonia nitrogen concentrations in lambs fed with low CP (13% on DM); however, they pointed out that this CP level ensured adequate nitrogen supply for ruminal microbial protein synthesis in hair sheep raised in a tropical environment.

In contrast, in our study, plasma creatinine concentration in the growing period did not differ between sampling hours, but in the finishing period it was higher at 8:00 a.m. and 12:00 p.m. than at 4:00 p.m., especially in the CP17 group. This could be explained by the postprandial variation in this catabolite in blood, maybe reflecting, in this study, a blood creatinine clearance and higher urinary turnover excretion before the last sampling (4:00 p.m.), which would follow the eating and drinking morning peak as they may affect the endogenous creatinine concentrations [39]. Accordingly, Vivian et al. [40] observed a decrease in blood creatinine concentration between 0 h and 12 h after feed intake in the finishing period of heavy lambs, which is in agreement with the present results. Consequently, the urea/creatinine ratio of the finishing period was higher at 4:00 p.m. than before, which would reflect a higher urea synthesis than creatinine in the last sampling, possibly linked to postprandial digestion. Other works have observed that blood creatinine concentration usually had few diurnal fluctuations (6:00 a.m. to 6:00 p.m.), but it was higher during diurnal than nocturnal sampling periods [41].

## 5. Conclusions

Overall, a single sample of faeces and blood could be used to assess OM, CP and P apparent digestibility, as well as urea and creatinine in light lambs fed ad libitum, and they were not affected by the interplay between sampling time and dietary CP level, although the CP digestibility estimate may be more affected by dietary CP in the morning (8:00 a.m.) during the growing period.

## Figures and Tables

**Table 1:** Dietary composition in the growing and finishing periods.

Item	Dietary Crude Protein		Barley Straw
	CP20/19	CP18/17	
**Growing period**			
OM (%)	94.3	93.9	94.8
CP (%, N × 6.25)	20.7	18.4	2.36
P (%)	0.42	0.45	0.07
AIA (%)	0.61	0.58	2.60
**Finishing period**			
OM (%)	94.5	94.5	95.6
CP (%, N × 6.25)	19.1	17.5	2.08
P (%)	0.42	0.42	0.07
AIA (%)	0.61	0.51	2.60

Note: %, on DM basis, unless otherwise stated. OM, Organic Matter; CP, Crude Protein; N, Nitrogen; P, Phosphorous; AIA, Acid-Insoluble Ash.

**Table 2:** Faecal composition in the growing and finishing periods.

Item	Sampling Time				Dietary Crude Protein			*p*-Value	
	8:00 a.m.	12:00 p.m.	4:00 p.m.	SD	CP20/19	CP18/17	SD	Hour	Diet
**Growing period**									
DM (%, on fresh-weight basis)	34.9	34.1	34.5	0.74	34.3	34.7	0.87	0.551	0.768
OM (%)	87.4	87.1	87.5	0.28	87.3	87.4	0.29	0.460	0.721
CP (%, N × 6.25)	21.3	20.5	20.4	0.51	21.3	20.3	0.43	0.444	0.111
P (%)	0.84 ^a^	0.98 ^b^	0.91 ^b^	0.04	0.99 ^a^	0.83 ^b^	0.04	0.026	0.024
AIA (%)	2.39	2.43	2.36	0.05	2.28 ^a^	2.51 ^b^	0.05	0.491	0.019
**Finishing period**									
DM (%, on fresh-weight basis)	35.7 ^a^	37.2 ^a,b^	38.7 ^b^	1.05	36.5	37.9	1.30	0.009	0.429
OM (%)	87.6	88.3	87.9	0.34	88.2	87.7	0.40	0.130	0.358
CP (%, N × 6.25)	19.1	18.6	19.4	0.51	19.3	18.8	0.54	0.463	0.441
P (%)	0.80	0.78	0.79	0.04	0.82	0.76	0.05	0.835	0.479
AIA (%)	2.61	2.62	2.72	0.12	2.69	2.61	0.17	0.259	0.755

Note: %, on DM basis, unless otherwise stated. The interaction between hour and diet did not affect any variable (*p* > 0.05). Within each row and effect, the statistical differences between means are described by different letters (^a,b^). SD, standard error; DM, Dry Matter; OM, Organic Matter; CP, Crude Protein; N, Nitrogen; P, Phosphorous; AIA, Acid-Insoluble Ash.

## Data Availability

The data presented in this study are available on request form the corresponding author.
